# The risk of lymph node metastases and their impact on survival in patients with appendiceal neuroendocrine neoplasms: a systematic review and meta-analysis of adult and paediatric patients

**DOI:** 10.1007/s12020-019-02072-y

**Published:** 2019-09-06

**Authors:** Kosmas Daskalakis, Krystallenia Alexandraki, Evanthia Kassi, Marina Tsoli, Anna Angelousi, Athanasia Ragkousi, Gregory Kaltsas

**Affiliations:** 1 Department of Surgical Sciences, Uppsala University, Uppsala, Sweden; 2 1st Department of Propaupedic Internal Medicine, Endocrine Oncology Unit, Laiko Hospital, National and Kapodistrian University of Athens, Athens, Greece; 3 Department of Biological Chemistry, Medical School, National and Kapodistrian University of Athens, Athens, Greece; 4 Clinical Sciences Research Laboratories, Warwick Medical School, University of Warwick, University Hospital, Coventry, UK; 5 Centre of Applied Biological & Exercise Sciences, Faculty of Health & Life Sciences, Coventry University, Coventry, UK

**Keywords:** Appendiceal neuroendocrine neoplasms, Locoregional lymph node metastases, Prophylactic right hemicolectomy

## Abstract

**Background:**

There are no clear histopathological parameters determining the risk of lymph node (LN) metastases and appropriateness of completion prophylactic right hemicolectomy (RHC) in patients with appendiceal neuroendocrine neoplasms (ANENs).

**Materials and methods:**

The PubMed, Cochrane Library, Embase, Web of Science and SCOPUS databases were searched up to November 2018. Quality/risk of bias was assessed using the Newcastle–Ottawa Scale (NOS).

**Results:**

A total of 526 articles were screened. In 11 adult and 3 paediatric studies, 602 and 77 unique patients, respectively, with ANEN and undergoing RHC, were included. The rate of LN metastases for a cutoff size >10 mm was 48.6% (vs 12.1% for lesions <10 mm) among adult patients, with an odds ratio (OR) of 4.8 (95% CI, 1.5–15.8). For 20 mm size cutoff, these figures were 61% (vs 28.2% for lesions <20 mm) with an OR of 3.2 (95% CI, 1.3–7.8). Vascular-, lymph vessel- and perineural invasions were identified as predictive factors for LN metastases in adult patients. In paediatric patients, there were no strong morphological predictors for LN metastases. The 10-year disease-specific survival (DSS) for adult patients without LN metastases was 99.2% vs 95.6% in patients with LN (OR: 0.2; 95% CI, 0.02–2.4). The complication rate of prophylactic RHC was 11.4%.

**Conclusions:**

This meta-analysis demonstrates that tumour size >20 mm as well as >10 mm and/or vascular-, lymph vessel- and perineural invasions are associated with increased risk for LN metastases in adult patients with ANEN. The prognostic value of LN positivity remains to be determined in further studies with long-term follow-up.

## Introduction

Appendiceal neuroendocrine neoplasms (ANENs) arise from the subepithelial neuroendocrine cells of the appendiceal wall and are generally discovered incidentally at appendectomy for acute appendicitis [1, 2]. They commonly exhibit a benign clinical course with a minority developing locoregional lymph node (LN) metastases. Few cases have been reported with distant stage disease [3]. The literature shows great diversity in terms of disease-specific mortality and its association with the presence of locoregional metastatic disease in LNs of the mesentery, as well as the type and extent of surgery undertaken. This diversity is mainly attributed to the fact that in the majority of series, not only well-differentiated (WD) ANENs were included [4, 5].

Due to the indolent course of the disease and because there is currently no adjuvant therapy that is known to improve overall survival (OS) in ANENs, surgery is considered the mainstay of treatment. Appendectomy alone has traditionally been considered an adequate treatment for ANENs <10 mm, whereas a completion prophylactic right hemicolectomy (RHC) following appendectomy is generally advocated for lesions >20 mm. This approach to ANEN management is also in accordance with current European Neuroendocrine Tumour Society (ENETS) guidelines [6]. However, histopathological parameters determining the risk of LN metastases and their associated mortality in adult and paediatric patients with an ANEN measuring between 10 and 20 mm have not been clearly defined and are still a matter of debate.

Earlier systematic reviews, including a recent appraisal from ENETS on unmet needs in ANEN management included only retrospective, observational, institutional and registry-based studies, as no randomized trial on ANENs is available to date [7]. Importantly, several large studies on this topic were published during recent years contributing valuable new evidence in the field of ANENs. To date, predominant predictive factors associated with disease outcomes in terms of locoregional metastatic propensity are size and grade of the primary tumour as defined by the Ki-67 proliferation index. Some investigators also argue the clinical importance of serosal penetration, i.e. meso-appendix invasion and other histopathological parameters.

Although the various ANEN size cut‐offs for completion prophylactic RHC used in recent publications have potentially caused a loss of valuable information, light has been shed on the predictive value of other histopathological factors of the appendectomy specimen with respect to the risk for locoregional LN metastases. There remains a great need for summarized evidence to address the “grey zones” in ANEN management and in particular appropriateness, effectiveness and safety of completion prophylactic RHC in ANEN cases. Foremost, the risk of LN metastases as a function of tumour size and their morphological characteristics remains to be determined.

Our aim was to compare the rate of LN metastases and their impact on survival for adult and paediatric patients with ANENs undergoing RHC at different size cutoffs, with and without the presence of specific morphological parameters at histopathology, and also to assess the rate of complications following RHC in ANENs.

## Patients and methods

### Study selection

National registry studies, along with retrospective cohort studies on ANEN in adult and paediatric patients undergoing surgery, were eligible for inclusion. The outcomes that were required for eligibility included at least two of the following terms: tumour size, location, grade, meso-appendix invasion, vascular invasion, lymph vessel invasion, perineural invasion and LN metastases, OS and complications. A sample size of at least ten ANEN patients undergoing RHC was necessary for study inclusion, at least in the adult ANEN population. Studies reporting data on gobbler cell appendiceal tumours and tumours of mixed histopathology together with WD ANEN were excluded. Only the latest eligible study was selected among multiple reports from the same research group, institution or national registries, e.g. the SEER database. In cases with overlap in patient cohorts of two studies, the most recent and largest study was included, unless data were available at the individual level, allowing for exclusion of duplicate cases. Evolving classifications in the histopathology of gastro-enteropancreatic NENs mainly concern pancreatic NENs and did not affect the selection of eligible studies. We followed Preferred Reporting Items for Systematic Reviews and Meta‐Analyses (PRISMA) guidelines for reporting [8]. No study protocol for this meta‐analysis was published or registered before the study was undertaken. The primary endpoint was to evaluate the risk for LN metastases using different size cutoffs and other morphological parameters at histopathology in order to select best adult and paediatric candidates for RHC. The secondary endpoints were to evaluate the impact of LN positivity and that of prophylactic RHC in survival. In particular the inclusion criteria included pathological ANEN diagnosis after surgery, a RHC specimen for histopathological evaluation and the presence of size 10 and 20 mm cutoffs as well as that of other morphological parameters at histopathology.

### Search strategy

To identify studies and determine eligibility, we conducted a systematic search in the PubMed, Cochrane Library, Embase, Web of Science and SCOPUS databases. Search terms included “appendiceal neuroendocrine tumour”, “appendectomy”, “surgery”, “resection”, “RHC” and “hemicolectomy”, which were all used in combination with the boolean operators AND and OR. The search terms were input as free text. All eligible titles and abstracts were assessed in duplicate by two of the authors (K.D. and K.A.). Full manuscripts were examined as necessary to finalize the study selection. Reference lists of studies reviewed for eligibility were also searched, to identify additional studies.

### Data extraction

Data were independently extracted by two of the authors (K.D. and K.A). The primary outcome was defined as the prevalence of LN metastases at RHC using different morphological parameters at histopathology. We defined the following secondary outcomes: mortality associated with LN metastases and complication rate occurring in ANEN patients undergoing prophylactic RHC. Potentially eligible studies with double zero cells of investigated outcomes were not included in the analysis. The study hypothesis was formulated before data collection. Any discrepancies concerning the extracted data between the two authors were resolved by consensus or by consultation of a third author (G.K.).

### Risk for bias

Our classification of observational institutional and registry-based studies followed classical epidemiologic study designs (e.g. case-control and cohort study), with the key element of this being based on analysis features [9]. For quality assessment of the cohort studies included we applied a score system and assessed the studies in accordance with the Newcastle–Ottawa Scale (NOS) criteria. The total score range was from 0 (worst) to 9 (best) for case-control and cohort studies, with a score of at least 6 suggesting high quality [10].

### Statistical analysis and exploration of heterogeneity

Statistical analyses were completed using STATA 14.0 software (StataCorp, 2015. Stata Statistical Software: Release 14. College Station, TX: StataCorp LP). We adopted a random-effects model to combine the summary statistics and reported pooled OR for primary and secondary outcomes. Statistical heterogeneity was evaluated by the I^2^ method and the χ^2^ test to calculate *p*-values; *I*^2^ values > 50% suggested increased heterogeneity. Potential publication bias and small study effects were assessed by visually inspecting funnel and Galbraith plots and conducting complementary tests (Egger’s and Harbord’s tests) as appropriate. We tested the small sample size effect in paediatric ANEN population using Begg test and trim and fill method to correct the ORs if necessary. In addition to principal analyses, random-effects univariate meta-regression analyses were performed to account for statistical interstudy heterogeneity as appropriate. The results were given as OR with 95% CI, and *p*-values, as appropriate. The 5% level (*p*-value < 0.05) was set to indicate statistical significance.

## Results

### Characteristics of included studies

**Fig. 1:**
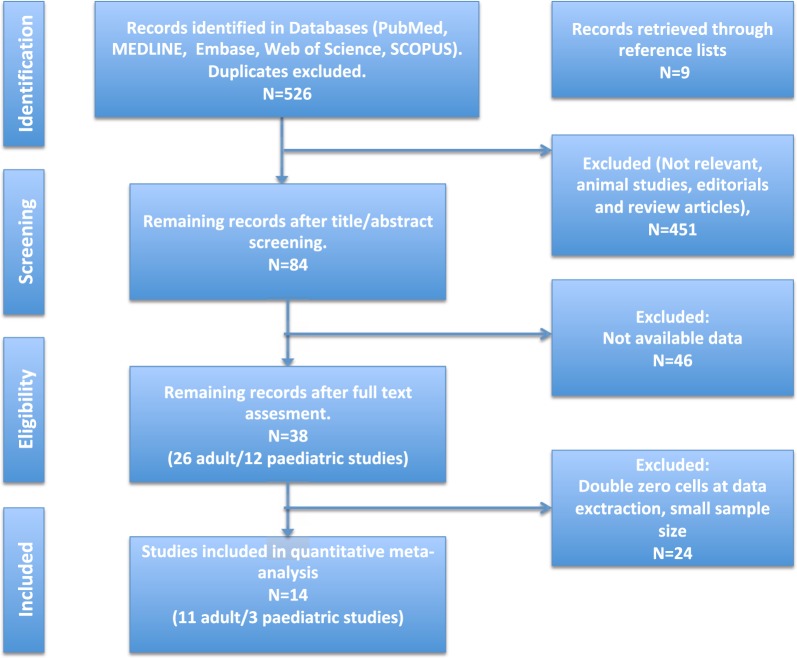
PRISMA flow diagram of study selection

A total of 526 articles were screened. From 11 adult and 3 paediatric studies, 602 and 77 unique patients with ANEN undergoing RHC were included, respectively. The literature search and the selection of included studies are presented in the PRISMA flow diagram (Fig. 1). The characteristics of the included studies are summarized in Table 1, including information on funding and potential conflict of interest.

**Table 1:** Characteristics of the included studies

	Study design	No of patients (undergoing RHC^a^)	Outcomes		Funding and conflict of interest statement
			Primary (positive LN^a^ status)	Secondary	
**Adult studies**					
Alexandraki et al. [11]	Single-centre retrospective cohort study	12	2/12 (data available at the individual level)	Median follow-up 2.1 years; complications 17%	No funding or conflict of interest reported
Galanopoulos et al. [16]	Single-centre retrospective cohort study	72	23/72 (data not available at the individual level)	Median follow up, 5- and 10-year DSS not reported	No funding or conflict of interest information reported
Sarshekeh et al. [12]	SEER^a^-based cohort study	194	106/194 (data not available at the individual level)	Median follow up not reported; 10-year DSS reported with respect to LN status	No funding or conflict of interest reported
Pawa et al. [3]	Multicenter retrospective cohort study	49	12/49 (data not available at the individual level)	Median follow-up 3.2 years; DSS not reported; complications 2%	No specific funding. Author support reported: Dr Heinz-Horst Deichmann Stiftung, ENETS Fellowship, Cancer Research UK and European Union FP7-MC-IEF funding scheme
Rault-Petit et al. [13]	National (French) registry-based retrospective cohort study (Recruitment from RENATEN and TENpath)	100	23/100 (data not available at the individual level)	Median follow-up 0.25 years; DSS not reported	Funding: This study received financial support from a Grant provided by GTE/ APTED and the French NET patient association. The authors report no conflicts of interest.
Steffen et al. [14]	Multicenter retrospective cohort study	10	1/10 (data not available at the individual level)	Median follow up 13.7 years; 5- and 10-year overall and relative survival were reported	No funding or conflict of interest reported
Woltering et al. [22]	Single-centre retrospective cohort study (conference paper)	Not reported	Not reported (data not available at the individual level)	Median follow up not reported; 10-year DSS was reported with respect to LN status	No funding or conflict of interest information mentioned in conference paper
Moertel et al. [19]	Single-centre retrospective cohort study	11	7/11 (data not available at the individual level)	Median follow up 26 years; 10-year DSS was reported with respect to LN status (100% in both arms)	No funding or conflict of interest information mentioned in article
Grozinsky-Glasberg et al. [15]	Multicenter retrospective cohort study	16	8/16 (data available at the individual level; duplicate data from one institution were removed)	Median follow up 3.6 years; DSS not reported.	No funding or conflict of interest reported
Brighi et al. [17]	Multicenter retrospective cohort study	69	21/69 (data not available at the individual level)	Median follow up and 5-year, 10-year DSS not reported	No funding or conflict of interest reported
Liu et al. [18]	Single-centre retrospective cohort study	37	10/37 (data not available at the individual level)	Median follow up and 5-year, 10-year DSS not reported	No funding or conflict of interest information mentioned in article
Kleiman et al. [20]	Single-centre retrospective cohort study	32	7/32 (data not available at the individual level)	Median follow up 1.5 years; DSS not reported	No funding or conflict of interest reported
**Paediatric studies**					
Boxberger et al. [29]	Multicenter prospective cohort study	60	9/60 (data not available at the individual level)	Mean follow up 2.9 years and 5-year, 10-year DSS not reported	Funding: Deutsche Kinderkrebsstiftung; W.A. Drenckmann Stiftung; Magdeburger Forderkreis krebskranker Kinder e.V. No conflict of interest reported
Wu et al. [28]	Single-centre retrospective cohort study	7	1/7 (data not available at the individual level)	Mean follow up 0.3 years; DSS not reported	No funding or conflict of interest reported
De Lambert et al. [30]	Multicenter retrospective cohort study	10	3/10 (data available at the individual level)	Mean follow up 0.3 years; DSS not reported	No funding or conflict of interest reported

^a^*RHC* right hemicolectomy, *LN* lymph nodes, *SEER* surveillance, epidemiology and end results, *DSS* disease-specific survival, *ANEN* appendiceal neuroendocrine neoplasm, *RENATEN* the national clinical network of NET, *TENpath* the national pathological network, *GTE/APTED* the French Endocrine Tumor Group (Groupe des tumeurs endocrine, *GTE*)

### Quality assessment and risk of bias within studies

The results of the quality assessment of each study are presented in Table 2 (NOS template). We identified no randomized trials. All studies were observational cohort studies based on retrospective analysis of institutional or registry data. The variety of studies included did not vary considerably. Factors contributing to lower NOS scores were small sample sizes, ambiguity over ANEN inclusion criteria, inadequate follow‐up and/or many patients lost to follow‐up, lack of clarity over criteria for completion prophylactic RHC and failure to report compliance and complications rates for patients undergoing RHC.

**Table 2:** Newcastle–Ottawa scale (NOS) cohort star template

	Selection	Comparability	Exposure
**Adult studies**			
Alexandraki et al. [11]	★★★	★★	★★
Galanopoulos et al. [16]	★★★	★★	★★
Sarshekeh et al. [22]	★★	★★	★★
Pawa et al. [3]	★★★	★★	★★
Rault-Petit et al. [13]	★★	★★	★★
Steffen et al. [14]	★★★	★★	★★★
Woltering et al. [22]	★★	★	★★
Moertel et al. [19]	★★★	★★	★★★
Grozinsky-Glasberg et al. [15]	★★★	★★	★★
Brighi et al. [17]	★★★	★★	★★
Liu et al. [18]	★★★	★	★★
Kleiman et al. [20]	★★★	★★	★★
**Paediatric studies**			
Boxberger et al. [29]	★★★★	★★	★★
Wu et al. [28]	★★★	★★	★★
De Lambert et al. [30]	★★★	★★	★★

To determine the risk of reporting bias and the presence of small study effects, effect size estimates from the included studies were plotted against the measure of each study’s size on funnel plots for each investigated parameter (Supplementary Figs. 1–14, Supplement). The visually observed asymmetry in the distribution of the funnel plots necessitated complementary tests that did not demonstrate small size effects (Supplementary Figs. 1–14, Supplement). Reasons for funnel plot asymmetry could be the fact that a small number of studies were included (<10 studies in several meta-analyses), heterogeneity between studies and publication bias.

### Pooled results for primary tumour size

**Fig. 2:**
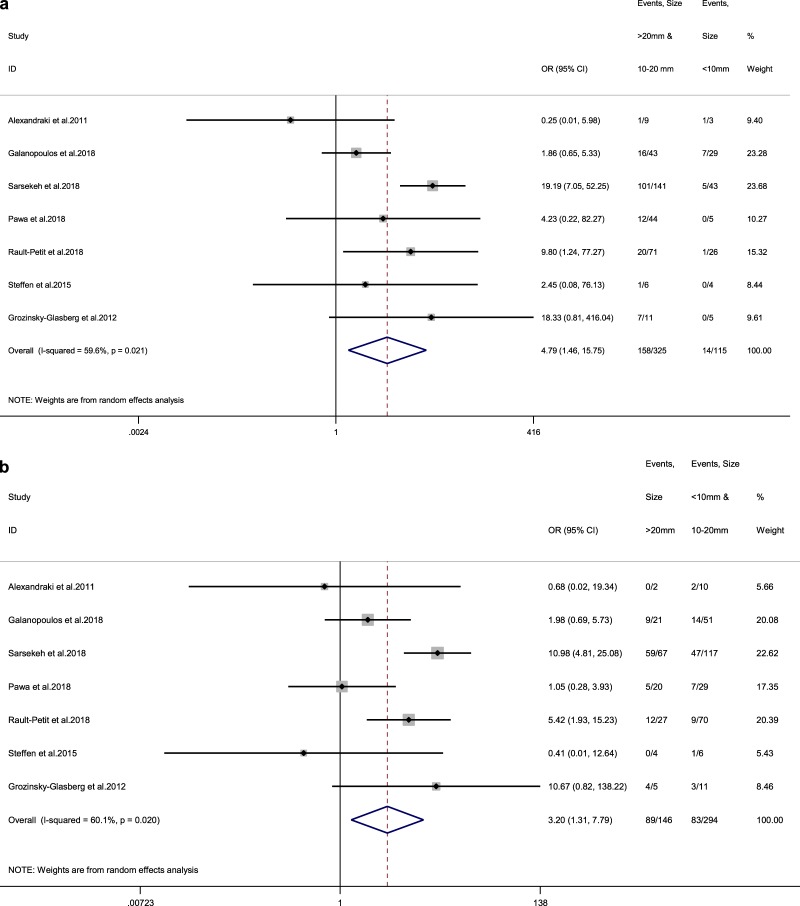
**a** Forest plot comparing the rate of LN metastases at RHC and at a 10 mm size cutoff, i.e. in tumour size >10 mm vs tumour size <10 mm. **b** Forest plot comparing the rate of LN metastases at RHC at a 20 mm size cutoff, i.e. in tumour size >20 mm vs tumour size <20 mm. Meta‐analysis of all studies carried out using a random‐effects model; Odds ratios are shown with 95% confidence intervals

We identified seven studies reporting LN status at RHC for tumour size cutoffs of 10 and 20 mm [3, 11–16]. The rate of LN metastases for a cutoff size >10 mm was 48.6% (vs 12.1% for lesions <10 mm) among adult patients, with a random-effects OR of 4.8 (95% CI, 1.5–15.8; heterogeneity, *P* = 0.061; *I*^2 ^= 46.3%, Egger’s *p*-value = 0.093, Fig. 2a). For a cutoff size of 20 mm, these figures were 61% (vs 28.2% for lesions <20 mm) with a random-effects OR of 3.2 (95% CI, 1.3–7.8; heterogeneity, *P* = 0.020; *I*^2^ = 60.1%, Egger’s *p*-value = 0.036, Fig. 2b).

**Fig. 3:**
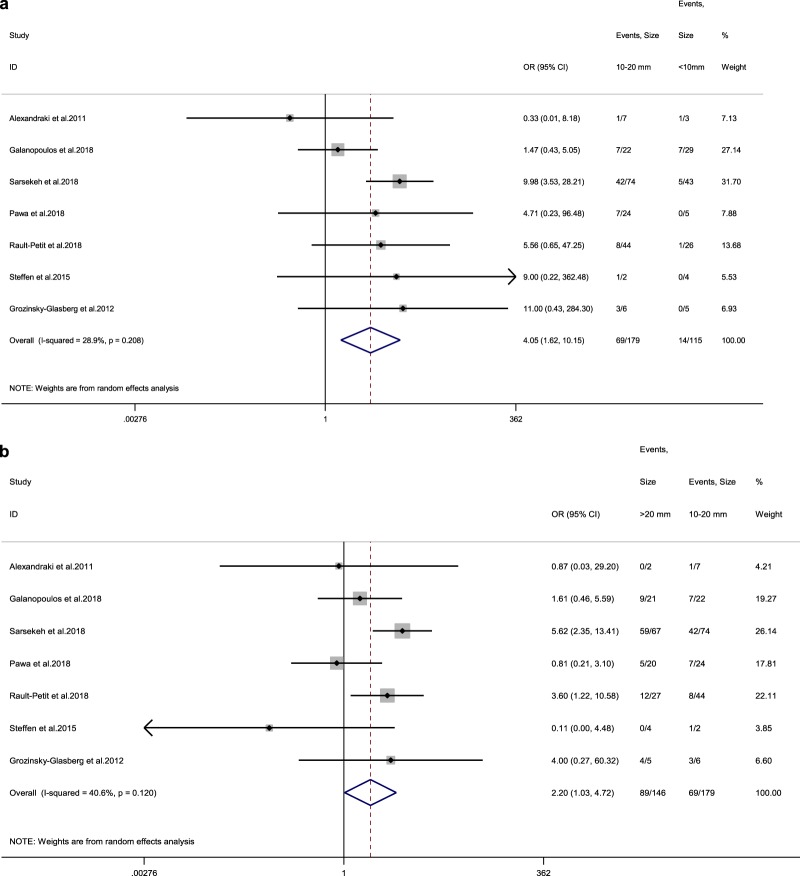
**a** Forest plot comparing the rate of LN metastases at RHC in tumours <10 mm vs tumours between 10–20 mm. **b** Forest plot comparing the rate of LN metastases at RHC in tumours >20 mm vs tumours between 10–20 mm. Meta‐analysis of all studies carried out using a random‐effects model; Odds ratios are shown with 95% confidence interval

We also performed subgroup analysis for tumour sizes of 10–20 mm vs <10 mm and 10–20 mm vs >20 mm separately, to address the grey zone of 10–20 mm in ANEN management and avoid contamination of this subgroup by larger and smaller tumours, respectively. The rate of LN metastases for <10 mm was 12.1% vs 38.5% for lesions 10–20 mm vs 61% for >20 mm among adult patients, with a random-effects OR of 4.1 for the first comparison (95% CI, 1.6–10.2; heterogeneity, *P* = 0.208; *I*^2^ = 28.9%, Egger’s *p*-value = 0.120 Fig. 3a) and of 2.2 for the second one (95% CI, 1–4.7; heterogeneity, *P* = 0.120; *I*^2^ = 40.6%, Egger’s *p*-value = 0.031, Fig. 3b).

### Pooled results for tumour location

**Fig. 4:**
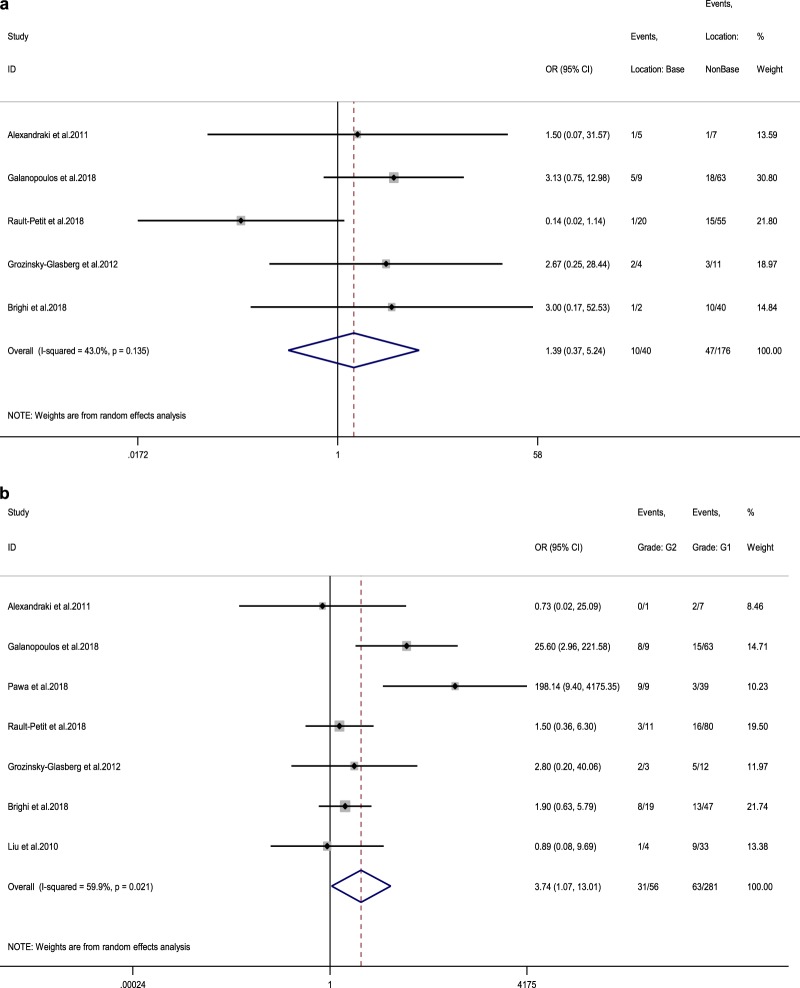
**a** Forest plot comparing the rate of LN metastases at RHC in tumours located in the appendix base vs tumours located in the body or tail of the appendix. **b** Forest plot comparing the rate of LN metastases at RHC in Grade 1 vs Grade 2 ANEN. Meta‐analysis of all studies carried out using a random‐effects model; Odds ratios are shown with 95% confidence intervals

We identified five studies reporting LN status at RHC in connection to ANEN location on the appendix (base vs non-base) [11, 13, 15–17]. The rate of LN metastases in ANENs located at the base of the appendix was 25% (vs 26.7% for lesions in the body or apex of the appendix) among adult patients, with a random-effects OR of 1.4 (95% CI, 0.4–5.2; heterogeneity, *P* = 0.135; *I*^2^ = 43%, Egger’s *p*-value = 0.663 Fig. 4a).

### Pooled results for tumour grade

Seven studies reporting LN status at RHC relating to WD ANEN grade (Grade 1 [G1] vs Grade 2 [G1]) were included in this analysis [3, 11, 13, 15–18]. The rate of LN metastases in G1 ANEN was 22.4% (vs 55.4% for G2 lesions) among adult patients, with a random-effects OR of 3.7 (95% CI, 1.1–13; heterogeneity, *P* = 0.021; *I*^2^ = 59.9%; Egger’s *p*-value = 0.947; Fig. 4b).

With respect to the observed high interstudy heterogeneity in grade analysis, when considering potential effect modifiers in univariate metagression analysis, there were significant effects of size and other morphological parameters (tumour size 10–20 mm *P* > |*z*|: 0.012; location, base *P* > |*z*|: 0.026; meso-appendiceal invasion *P* > |*z*|: 0.044; lymph vessel invasion *P* > |*z*|: 0.039; perineural invasion *P* > |*z*|: 0.049), suggesting that more than one parameters may impact the risk for LN positivity in Grade 2 patients, e.g. in the grey zone of 10–20 mm tumour size.

### Pooled results for other morphological parameters (meso-appendix, vascular, lymph vessel and perineural invasion)

**Fig. 5:**
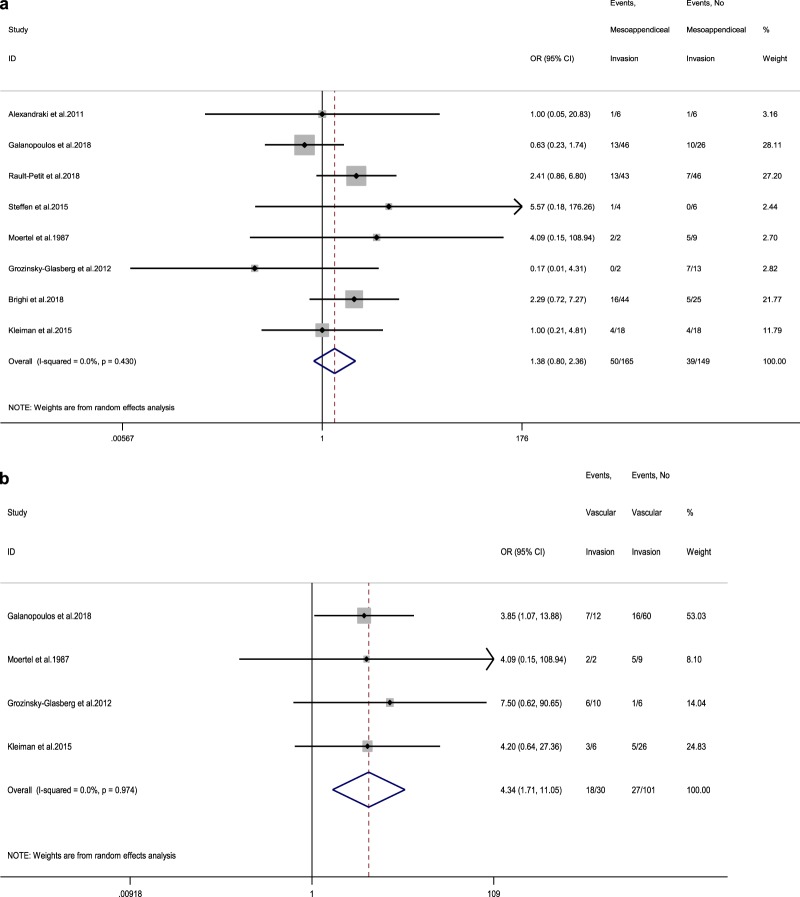
**a** Forest plot comparing the rate of LN metastases at RHC in tumours with meso-appendiceal invasion vs tumours without invasion of the meso-appendix. **b** Forest plot comparing the rate of LN metastases at RHC in tumours with vascular invasion vs tumours without vascular invasion. Meta‐analysis of all studies carried out using a random‐effects model; Odds ratios are shown with 95% confidence interval

Seven studies reporting LN status at RHC in connection to meso-appendix invasion were included [11, 13, 14, 16, 17, 19, 20]. The rate of LN metastases in adult patients demonstrating meso-appendix invasion was 30.3% (vs 26.2% in adult patients without meso-appendix invasion), with a random-effects OR of 1.4 (95% CI, 0.8–2.4; heterogeneity, *P* = 0.43; *I*^2^ = 0%, Egger’s *p*-value = 0.607, Fig. 5a).

**Fig. 6:**
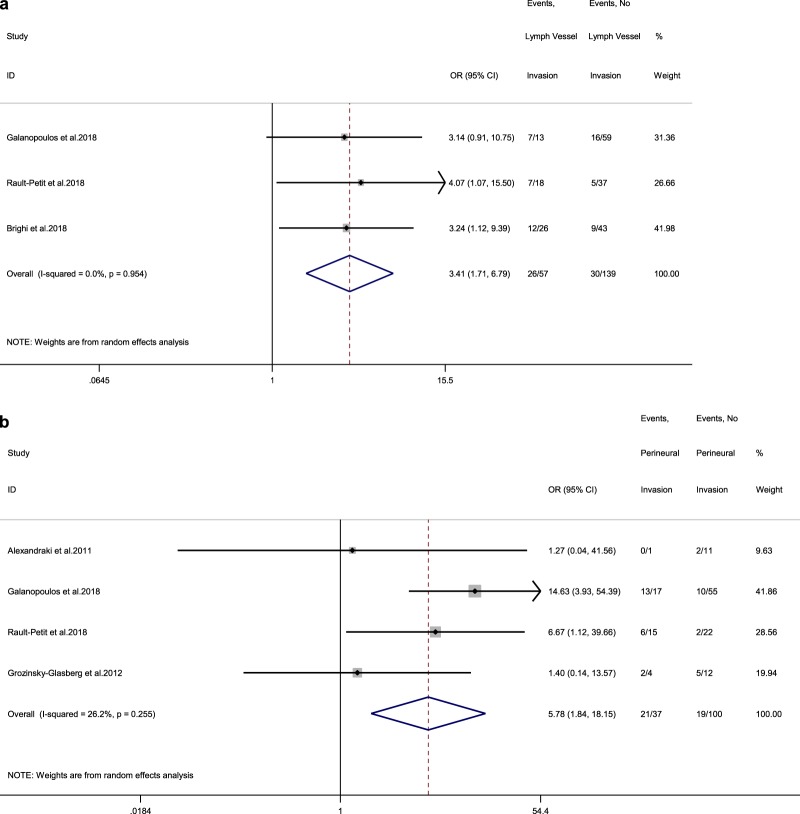
**a** Forest plot comparing the rate of LN metastases at RHC in tumours with lymph vessel invasion vs tumours without lymph vessel invasion. **b** Forest plot comparing the rate of LN metastases at RHC in tumours with perineural invasion vs tumours without perineural invasion. Meta‐analysis of all studies carried out using a random‐effects model; Odds ratios are shown with 95% confidence interval

In addition, we identified four adult studies reporting LN status at RHC in connection to vascular invasion [15, 16, 19, 20]. The rate of LN metastases in these patients was 60% (vs 26.7% in patients without vascular invasion), with a random-effects OR of 4.3 (95% CI, 1.7–11; heterogeneity, *P* = 0.974; *I*^2^ = 0%, Egger’s *p*-value = 0.095, Fig. 5b). For other morphological parameters, such as lymph vessel and perineural invasion, we identified three [13, 17, 21] and four eligible adult studies [11, 13, 15, 16], respectively. The rate of LN metastases in patients demonstrating lymph vessel invasion was 45.6% (vs 21.6% in patients without lymph vessel invasion), with a random-effects OR of 3.4 (95% CI, 1.7–6.8; heterogeneity, *P* = 0.954; *I*^2^ = 0%, Egger’s *p*-value = 0.690, Fig. 6a). These figures for ANEN with perineural invasion were: LN metastases rate 56.8% (vs 19% in patients without perineural invasion) with a random-effects OR of 5.8 (95% CI, 1.8–18.2; heterogeneity, *P* = 0.255; *I*^2^ = 26.2%, Egger’s *p*-value = 0.065, Fig. 6b).

### Pooled results for disease-specific survival (DSS) and complications

**Fig. 7:**
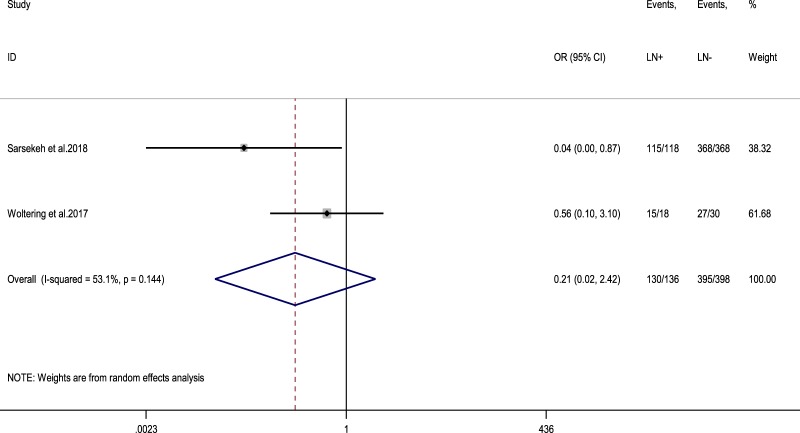
Forest plot comparing 10-year disease-specific survival in ANEN patients with LN metastases vs ANEN patients without LN metastases. Meta‐analysis of all studies carried out using a random‐effects model; Odds ratios are shown with 95% confidence intervals

Only two studies reported 10-year DSS stratified by LN status at RHC and these two were meta-analysed [12, 22]. The 10-year DSS rate for adult patients without LN metastases was 99.2% compared with 95.6% for patients with LN undergoing RHC (random-effects OR of 0.2, 95% CI, 0.02–2.4; heterogeneity, *P* = 0.144; *I*^2^ = 53.1%, Fig. 7). In seven studies reporting 5-year DSS rates following RHC versus appendectomy alone, 100% DSS was demonstrated in both arms (RHC vs appendectomy alone) [14, 19, 23–26].

The rate of complications that was encountered in patients undergoing prophylactic RHC was reported in four studies [3, 11, 25, 27] and was as high as 11.4%. However, a lower complication rate was demonstrated over time when comparing the studies by the year of publication (chi-square, *P* < 0.0001). The complications’ severity ranged significantly, although it’s range was not appropriately classified.

### Pooled results for morphological parameters in paediatric patients

**Fig. 8:**
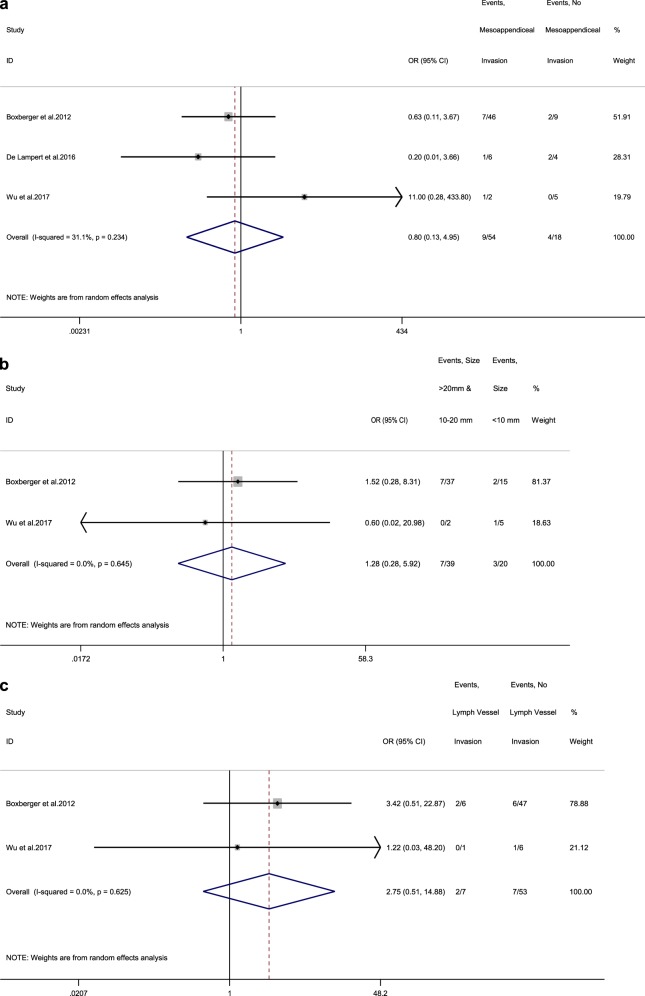
**a** Forest plot comparing the rate of LN metastases at RHC and at a 10 mm size cutoff, i.e. in tumour size >10 mm vs tumour size <10 mm for paediatric patients. **b** Forest plot comparing the rate of LN metastases at RHC in tumours with meso-appendiceal invasion vs tumours without invasion of the meso-appendix in paediatric patients. **c** Forest plot comparing the rate of LN metastases at RHC in tumours with lymph vessel invasion vs tumours without lymph vessel invasion in paediatric patients. Meta‐analysis of paediatric studies carried out using a random‐effects model; Odds ratios are shown with 95% confidence interval

The rate of LN metastases for a cutoff size >10 mm was 17.9% (vs 15% for lesions <10 mm) among paediatric patients, with a random-effects OR of 1.3 (95% CI, 0.3–5.9; heterogeneity, *P* = 0.645; *I*^2^ = 0%, Begg’s *p*-value = 0.317, Supplementary Fig. 8A) [28, 29]. For meso-appendix invasion, the rate of LN metastases was 16.7% in patients with this invasion (vs 22.2% in patients without), with a random-effects OR of 0.8 (95% CI, 0.1–5; heterogeneity, *P* = 0.234; *I*^2^ = 31.1%; Egger’s *p*-value = 0.628; Begg’s *p*-value = 0.602; Fig. 8b) [28–30]. Finally, the rate of LN metastases was 28.6% in paediatric patients with lymph vessel invasion (vs 13.2% in patients without), with a random-effects OR of 2.8 (95% CI, 0.5–14.9; heterogeneity, *P* = 0.625; *I*^2^ = 0%; Begg’s *p*-value = 0.317; Fig. 8c) [28, 29]. The trim and fill methods were not applied due to lack of heterogeneity in the included paediatric studies. In three studies reporting 10-year DSS rates following RHC vs appendectomy alone, 100% DSS was demonstrated in both arms (RHC vs appendectomy alone) [31–33]. In addition, three more studies were identified reporting 5-year DSS of 100% irrespective of the extent of the surgical procedure undertaken [34–36].

Generally, in paediatric studies, available data were limited and not always appropriately reported. Meta-analysis was not feasible for tumour size cutoff >20 mm as only one study was eligible (OR = 6.080, 95% CI: 1.213–30.473) [29]. One study only addressed grade (OR: 1.889, 95% CI: 0.050–72.022) and vascular invasion (OR: 0.375 95% CI: 0.022–6.348) in paediatric patients [30]. Data on complications secondary to prophylactic RHC were not available.

## Discussion

The present meta-analysis confirms that tumour size, vascular invasion, lymph vessel invasion and perineural invasion are strong predictors for LN metastases in adult patients with ANEN. On the contrary, tumour location on the appendix as well as meso-appendix invasion were not unambiguously confirmed as affecting the risk for LN metastases. Regarding different tumour size cutoffs in adult patients, the rate of LN metastases at RHC for lesions <10 mm was 12.1% vs 38.5% for lesions 10–20 mm vs 61% for tumours >20 mm. A random-effects OR of 4.8 (95% CI, 1.5–15.8) was estimated for a 10 mm size cutoff, whereas for a 20 mm cutoff, random-effects OR was 3.2 (95% CI, 1.3–7.8). Thus, primary tumour size retains its value as a predictive marker of metastatic propensity to locoregional LN.

Disease-specific mortality rates at 10 years of follow-up reported in two studies of adults with ANEN were as low as 0.8% and 4.4% for patients with and without LN metastases undergoing RHC, respectively. Notably, the presence of LN metastases does not seem to clearly affect survival in patients who have undergone curative resection, i.e. either RHC or appendectomy alone. Thus, we were unable to provide evidence that the presence of LN metastases affects OS or that RHC exerts a prophylactic effect. The complication rate of prophylactic RHC in this meta-analysis was 11.4%, with lower complication rates demonstrated over time, implying a generally safer procedure nowadays. In paediatric patients, available data were limited and no clear relationships between the parameters we investigated and LN metastases were evident. However, regardless of the extent of surgery in children, favourable long-term survival rates were reported, implying an excellent prognosis for paediatric ANEN cases subjected to curative resection.

In the efforts to adopt a precision medicine surgical and post-treatment surveillance approach in the management of ANEN, one of the main challenges is related to the paucity of validated biomarkers to guide the extent of locoregional resective surgery, i.e. to select the best candidates for prophylactic RHC and monitor patient outcomes. Overall, the rate of LN metastases in our study prior to different strata division and analysis, in both adult and pediatric patients, was 34.6%, as compared with the corresponding figure of 24.5% in a recent meta-analysis in ANENs [37]. The reasons for this discrepancy may be multifaceted and could potentially imply that the implementation of not validated risk factors, such as meso-appendiceal invasion in the grey zone of 10–20 mm tumour size to guide the extent of surgery may have led to unnecessary completion RHCs.

We conducted an NOS quality assessment on all the included studies (Table 2). All cohort studies scored at least five points, achieving moderate to high quality. Heterogeneity between studies was observed in the assessment of certain parameters, i.e. lesion size cutoff of 20 mm, grade and mortality rate. As statistically significant “positive” results are more likely to be published rapidly in English language and high impact journals, leading to reporting biases, we also assessed non-English studies, as well as unpublished data from conference papers [21, 22]. Complementary testing did not reveal small study effects in the present meta-analysis. Further factors, such as confounding and the precision of measurements, e.g. ICD-coded data in registry-based studies, might have introduced bias [38]. Importantly, although registry-based studies might differ regarding the strength of their evidence, they still constitute a valuable information source for evidence-based medicine [9].

With regard to the tumour size cutoffs investigated in this meta-analysis, we should clarify that traditionally a tumour size >20 mm constitutes a general indication for more extensive surgery, commonly completion RHC, based on the seminal paper in the New England Journal of Medicine published by pioneers in the field, namely Moertel et al. in 1987 [19]. To date, for ANENs between 10 and 20 mm, various morphological parameters at histopathology have been applied as per ENETS guidelines to identify patients who would potentially benefit from completion prophylactic RHC. Therefore, for lesions <20 mm subjected to RHC, one or more morphological parameter was presumably present at histopathology in order to qualify for completion surgery. However, few studies reported patient data at the individual level and in some there was ambiguity over criteria for completion prophylactic RHC. Thus, the rates and OR of LN metastases in ANEN < 20 mm must be interpreted in the light of this knowledge.

The prognostic significance of meso-appendix invasion was not confirmed in our study. In the current ENETS guidelines, a meso-appendix invasion depth of >3 mm is arbitrarily used to distinguish T2 from T3 tumours in the TNM classification. However, there is insufficient information in the literature to substantiate whether meso-appendix invasion and the 3 mm cutoff are validated prognostic factors to guide the extent of surgery. In addition, the location of the primary tumour in the appendix has been implied as a risk factor for non-radical resection at appendectomy, thus metastatic propensity necessitating completion RHC. However, this was not confirmed in our study, as no clear association with the presence of LN metastases was evident. Finally, Grade 2 was potentially linked to higher risk for LN metastases; however, due to high interstudy heterogeneity and potential effect modifiers evident in metagression analysis, no safe conclusions could be derived. On the contrary, vascular, lymph vessel and perineural invasion were substantiated as strong predictors for LN metastases in adult patients with ANEN.

ANEN’s clinical course in children may be completely benign, as paediatric studies report 5‐ and 10-year DSS of 100% in all patients who had undergone curative resection, i.e. appendectomy alone or completion RHC. However, due to insufficient data having been reported, our meta-analysis could not confirm predictive factors for LM metastases to guide the extent of surgery as in adult patients. For tumour size cutoff >20 mm, a single study was eligible and reported an OR as high as 6.1, (95% CI: 1.2–30.4) for LN metastases. However the clinical significance of this remains unclear and should be validated in more studies [29].

Our study has some limitations. Importantly, it constitutes an unplanned subset analysis of multiple observational studies on the rare entity of ANEN. The eligible institutional studies were underpowered or not even designed to assess differences in the outcomes of our interest. Further limitations include a lack of centralized pathology review that not only concerns interstudy concordance for tumour size and other histopathological parameters, but also changes within the NEN grading system over time. Moreover, regarding the SEER data quality, it should be noted that complete pathological data were not routinely included in the SEER database prior to 1988. However, in the SEER report by Sarshekeh included in this meta-analysis study, participants were only eligible for inclusion after 1988. All these limitations concern the general applicability of older studies nowadays, yet robust effects (e.g. for vascular invasion and the 20 mm size cutoff) had already been observed in the seminal study by Moertel et al. 32 years ago, as well as in very recent reports. Another important limitation was the high interstudy heterogeneity encountered when assessing certain morphological parameters, i.e. grade. However, in the absence of prospective studies, we used the best available evidence and applied a comprehensive search strategy and advanced statistical methods, including meta-regression, demonstrating the risk of LN metastases in relation to histopathology and their prognostic significance in adult and paediatric patients with WD ANEN.

In the random-effects model we adopted for 10-year DSS, *I*^2^ was 53%, suggesting that substantial variability in effect estimates on LN status is due to real study differences (heterogeneity). This is also evident from the wide scatter of effect estimates with broad CI that depict the uncertainty around this pooled estimate. Therefore, there is no strong evidence to date that positive locoregional LN metastases are associated with higher mortality. Hence, the true impact of LN status in ANEN, and whether a prophylactic surgical approach is beneficial, both need to be elucidated in further studies with long-term (>10 years) follow-up.

## Conclusions

Our study provides a systematic review and quantitative meta-analysis of ANEN summarizing all available evidence and confirming previously established prognostic factors. It also highlights areas of future research needed in the field. In addition, we confirmed that tumour size >20 mm, as well as >10 mm in the presence of vascular, lymph vessel or perineural invasion, are associated with an increased risk for LN metastases in adult patients with ANEN. In paediatric studies, available information is limited and no strong morphological predictors for LN metastases could be confirmed. ANENs have a generally favourable prognosis with rather low disease-specific mortality rates, as demonstrated in the adult studies included in the meta-analysis. Survival does not seem to be significantly depreciated after curative ANEN resection with either RHC or appendectomy alone. In addition, paediatric ANEN prognosis is excellent when subjected to curative resection. Longitudinal studies with >10 years follow-up are warranted to determine the true impact of LN status in patient survival from real-life data and whether a prophylactic surgical approach with completion RHC based on the findings of the present meta-analysis would be beneficial.

## Supplementary information

Supplementary Information
