# Supplementary material for: The risk of lymph node metastases and their impact on survival in patients with appendiceal neuroendocrine neoplasms: a systematic review and meta-analysis of adult and paediatric patients
Source: Endocrine. 2019 Sep 6;67(1):20–34. doi: 10.1007/s12020-019-02072-y (PMC6969007; doi:10.1007/s12020-019-02072-y)
Supplement: Supplementary file 1 — Supplementary Information [file 12020_2019_2072_MOESM1_ESM.pdf]

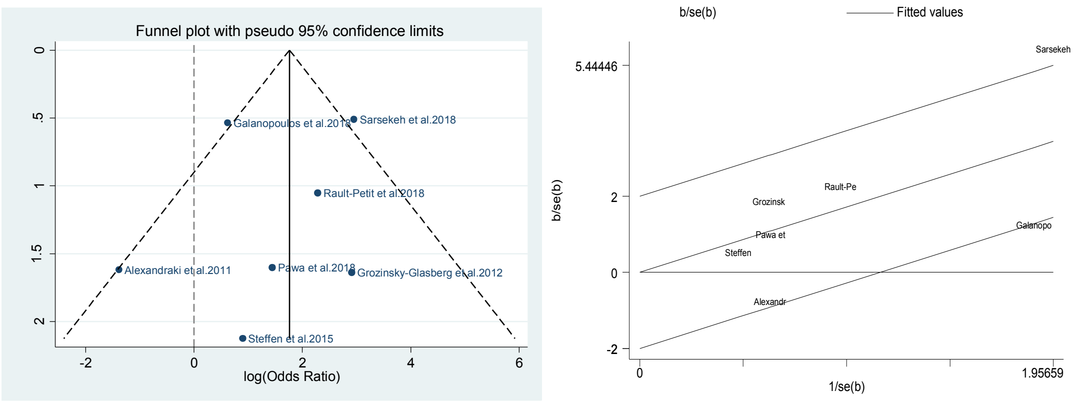

Supplementary Figure 1A: Funnel plot for studies included in the meta-analysis of tumour size cut-off at 10mm; 1B: Galbraith plot for studies included in this analysis.

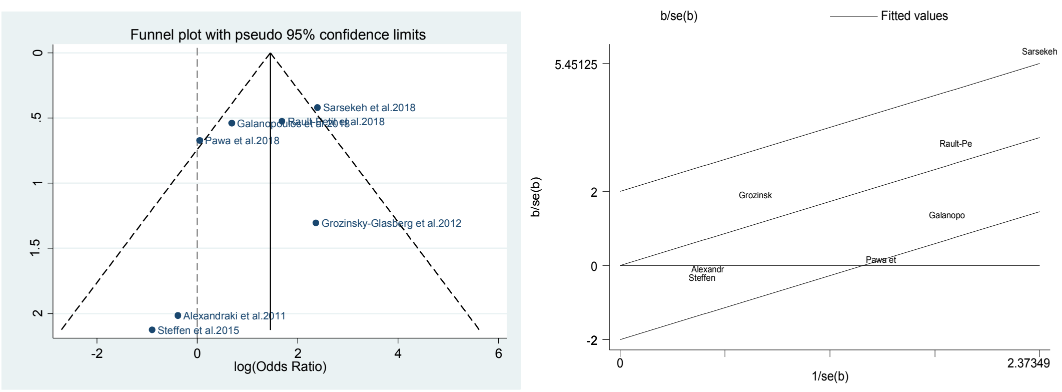

Supplementary Figure 2A: Funnel plot for studies included in the meta-analysis of tumour size cut-off at 20mm; 2B: Galbraith plot for studies included in this analysis.

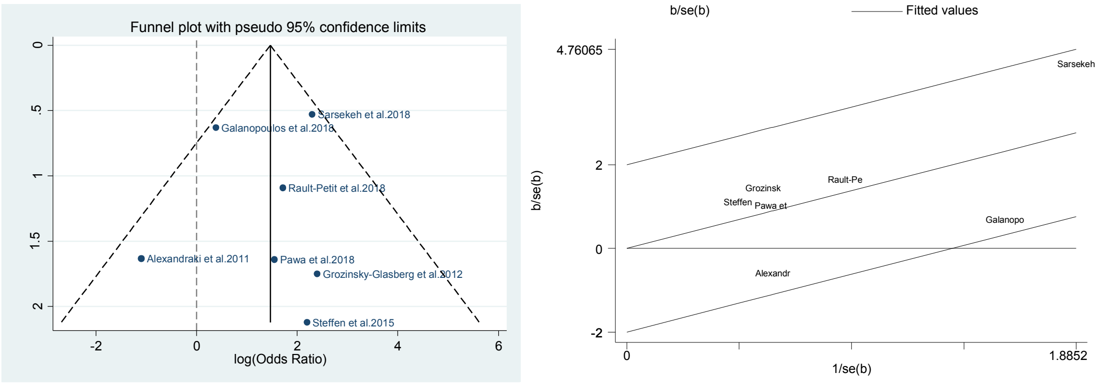

Supplementary Figure 3A: Funnel plot for studies included in the comparison of tumours 10-20mm vs tumours <10mm; 3B: Galbraith plot for studies included in this analysis.

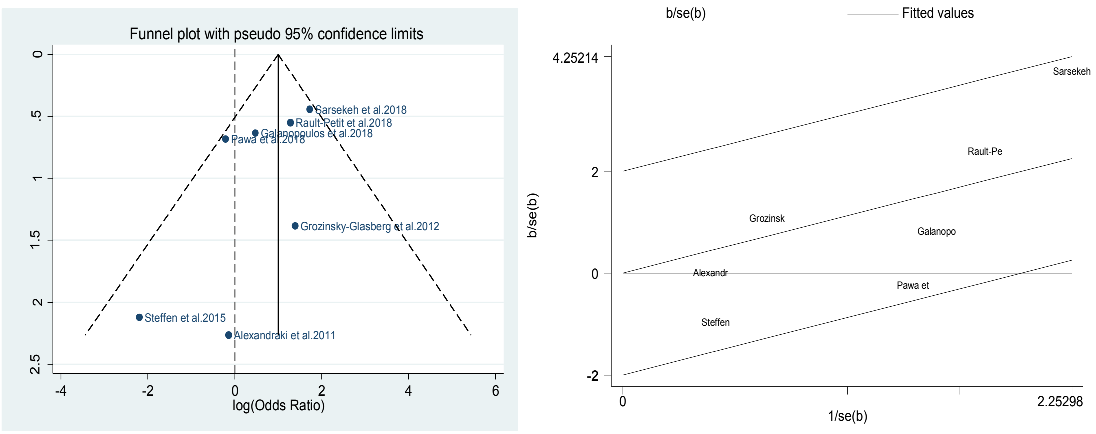

Supplementary Figure 4A: Funnel plot for studies included in the comparison of tumours 10-20mm vs tumours >20mm; 4B: Galbraith plot for studies included in this analysis.

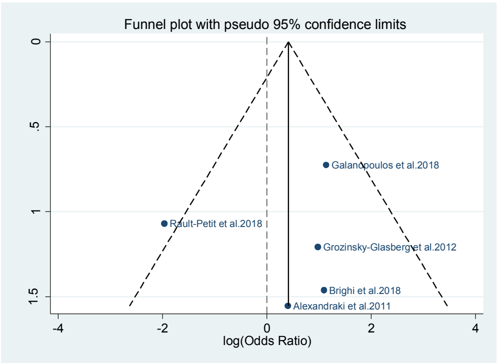

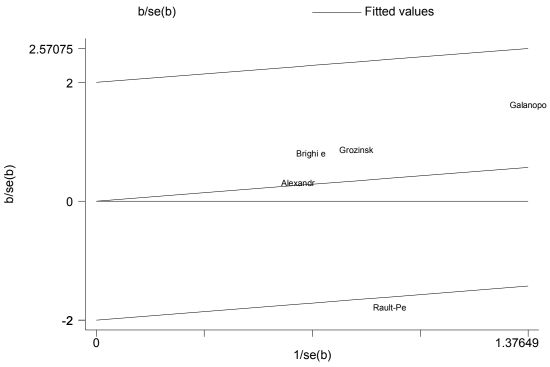

Supplementary Figure 5A: Funnel plot for studies included in the comparison of tumours at the appendix base vs tumours in the body or apex of the appendix; 5B: Galbraith plot for studies included in this analysis.

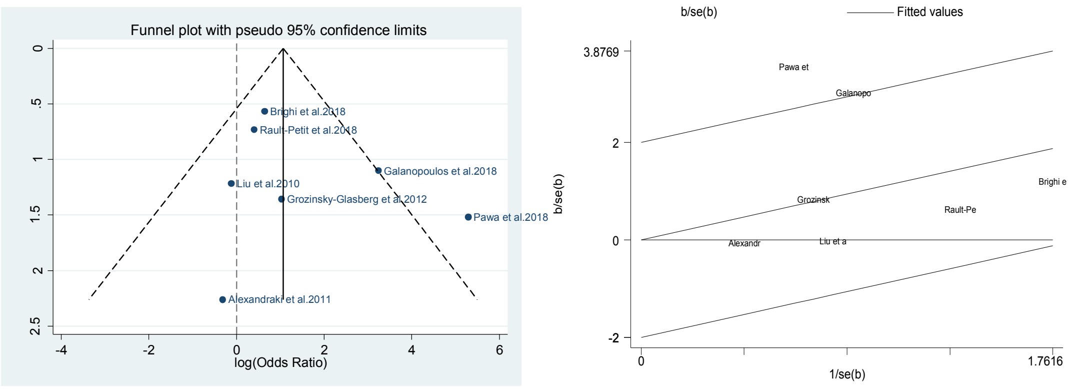

Supplementary Figure 6A: Funnel plot for studies included in the comparison of Grade 1 tumours vs Grade 2 tumours; 5B: Galbraith plot for studies included in this analysis.

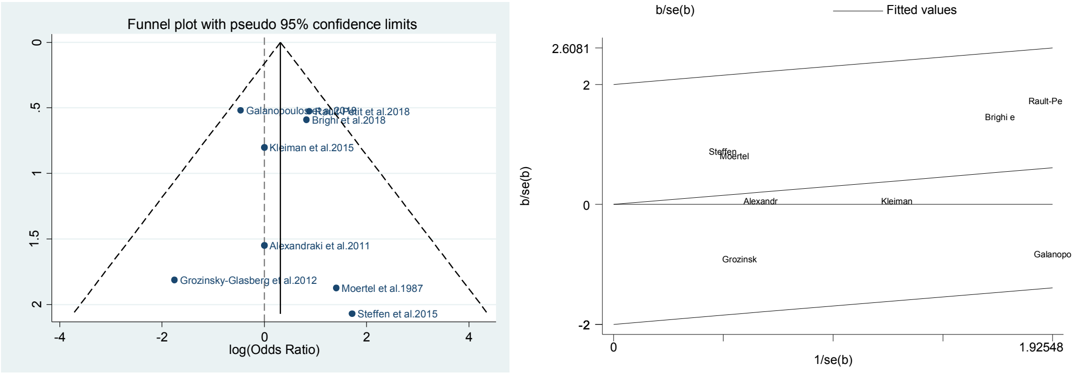

Supplementary Figure 7A: Funnel plot for studies included in the comparison of tumours with meso-appendix invasion vs tumours without meso-appendix invasion; 7B: Galbraith plot for studies included in this analysis.

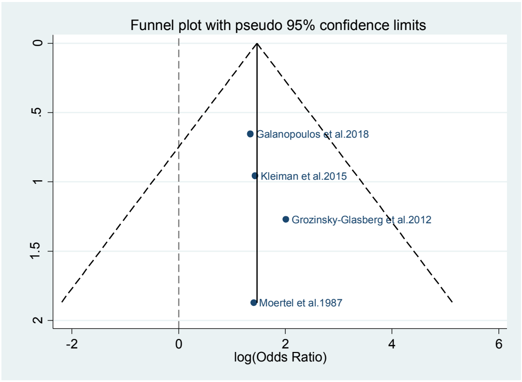

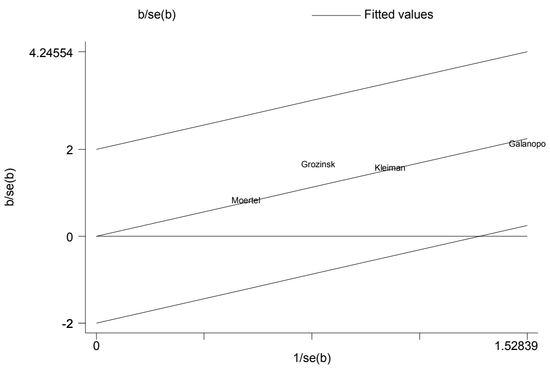

Supplementary Figure 8A: Funnel plot for studies included in the comparison of tumours with vascular invasion vs tumours without vascular invasion; 8B: Galbraith plot for studies included in this analysis.

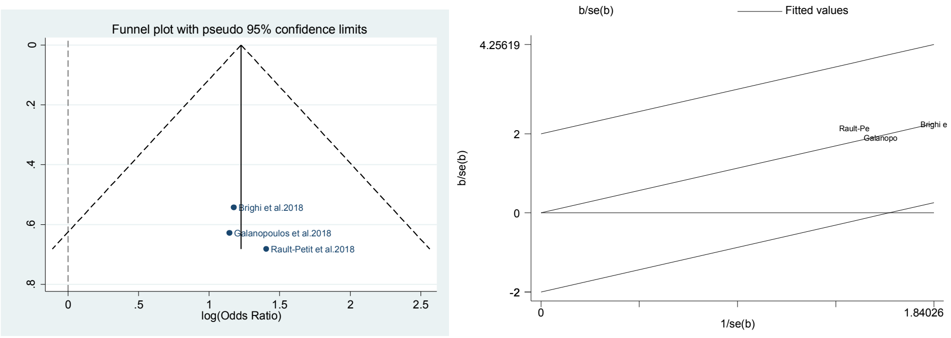

Supplementary Figure 9A: Funnel plot for studies included in the comparison of tumours with lymph vessel invasion vs tumours without lymph vessel invasion; 9B: Galbraith plot for studies included in this analysis.

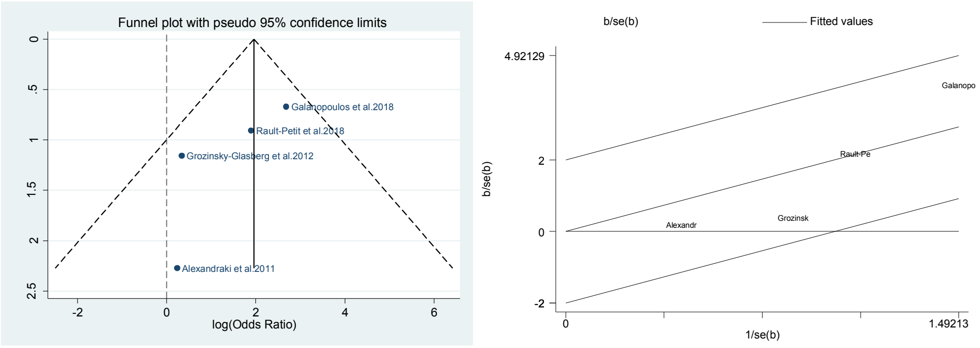

Supplementary Figure 10A: Funnel plot for studies included in the comparison of tumours with perineuaral invasion vs tumours without perineural invasion; 10B: Galbraith plot for studies included in this analysis.

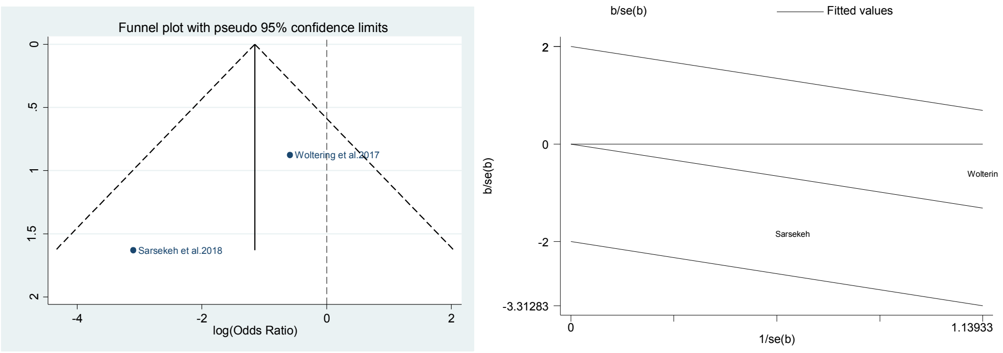

Supplementary Figure 11A: Funnel plot for studies included in the comparison of patients with lymph node metastases vs patients without lymph node metastases with respect to 10-year Disease Specific Survival; 11B: Galbraith plot for the two studies included in this analysis.

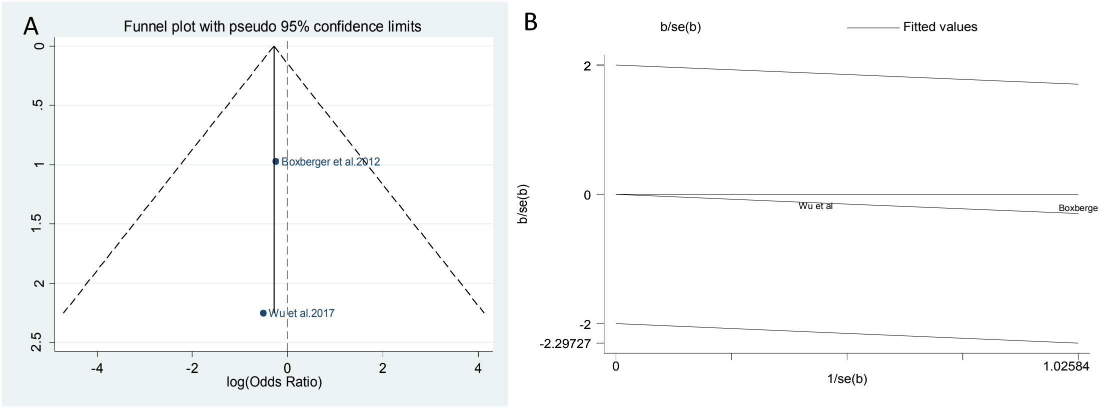

Supplementary Figure 12A: Funnel plot for studies included in the comparison of tumours >10mm vs tumours <10mm in paediatric studies; 12B: Galbraith plot for studies included in this analysis.

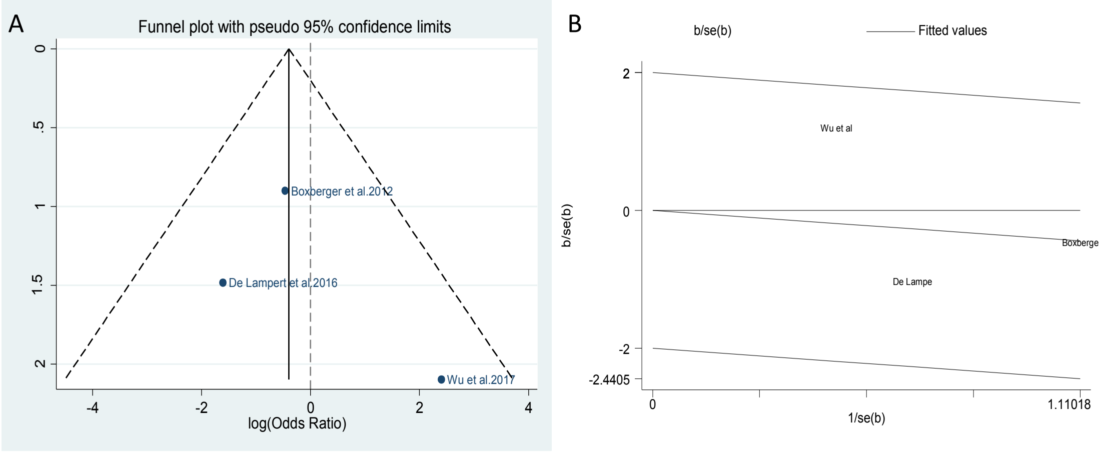

Supplementary Figure 13A: Funnel plot for studies included in the comparison of tumours with meso-appendix invasion vs tumours without meso-appendix invasion in paediatric studies; 13B: Galbraith plot for studies included in this analysis.

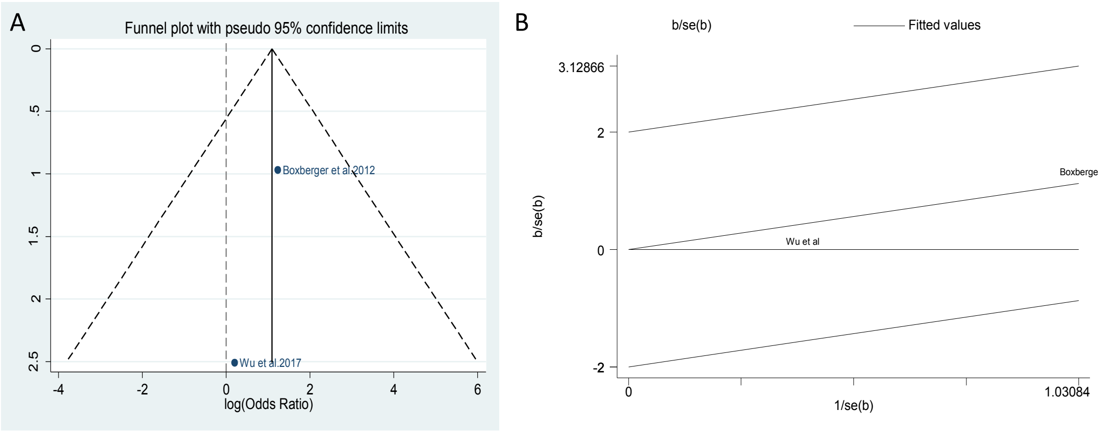

Supplementary Figure 14A: Funnel plot for studies included in the comparison of tumours with lymph vessel invasion vs tumours without lymph vessel invasion in paediatric studies; 14B: Galbraith plot for studies included in this analysis.
